# Antimicrobial or Subantimicrobial Antibiotic Therapy as an Adjunct to the Nonsurgical Periodontal Treatment: A Meta-Analysis

**DOI:** 10.5402/2012/581207

**Published:** 2012-10-22

**Authors:** Ana Patricia Moreno Villagrana, José Francisco Gómez Clavel

**Affiliations:** Carrera de Cirujano Dentista, FES-Iztacala, Universidad Nacional Autónoma de México, Tlalnepantla, MEX 54090, Mexico

## Abstract

The use of antibiotics in nonsurgical periodontal treatment is indicated in cases in which scaling and root planing present important limitations. However, their use is controversial due to the secondary effects associated with them and the disagreements regarding their prescription. The aim of this study is to determine the effectiveness of systemic antibiotics in the management of aggressive and chronic periodontitis. The study was based on a search of randomized, controlled clinical trials. Common data were concentrated and evaluated by means of an analysis of variance (ANOVA), and a meta-analysis of the results was performed. The meta-analysis (*P* < 0.05, 95% confidence interval, post hoc Bonferroni) determined that the supplementation of nonsurgical periodontal therapy with a systemic antibiotic treatment—amoxicillin with clavulanic acid and metronidazole or subantimicrobial dose doxycycline—provides statistically significant results in patients with aggressive or chronic periodontitis under periodontal treatment, whilst increasing the clinical attachment level of the gingiva and reducing periodontal probing depth.

## 1. Introduction

It is now recognized that the majority of connective tissue and bone destruction in periodontal tissues occurs indirectly as a result of an excessive immunoinflammatory response in the host to the presence of bacterial plaque in susceptible individuals. Although bacterial pathogens initiate the periodontal inflammation, the host response to these pathogens is equally if not more important in mediating connective tissue breakdown, including bone loss [1, 2]. The host-derived enzymes known as matrix-degrading metalloproteinases (MMPs) as well as changes in osteoclast activity driven by cytokines and prostanoids catalyze the breakdown of proteins, including collagen, gelatin, proteoglycan core protein, fibronectin, laminin, and elastin, located either on the cell plasma membrane or within the extracellular matrix [3–6]. Pathologically excessive levels of activity of the various MMPs degrade all of the major components of the extracellular matrix in the gingiva, the periodontal ligament, and the alveolar bone, including the collagen fibers (mostly type I and III), proteoglycans, ground substance, and basement membranes [7–9].

The standard treatment for periodontitis remains highly nonspecific, consisting of the mechanical debridement of the affected root surface in order to reduce the total bacterial load and change the environmental conditions of these microbial niches [19]. However, not all patients nor all sites respond uniformly and favorably to conventional mechanical therapy, and a small although relevant proportion of sites and patients do not respond adequately to this therapy. The reduced effectiveness of the therapy may be explained by a series of patient-related factors (local or generalized), the extent and nature of attachment loss, local anatomic variations, the form of the periodontal disease, and the composition and persistence of periodontal pathogens [20–22]. Based on this, numerous authors have hypothesized that purely mechanical treatment may not be effective for certain patients, that is, patients with aggressive forms of periodontitis or associated with predisposed medical conditions, in whom additional antimicrobial therapy would improve their clinical outcome and would even be essential for successful treatment [23, 24].

Several studies have evaluated the use of antibiotics to stop or reduce the progression of periodontitis. Systemically administered antibiotics penetrate the periodontal tissues via the serum. There, they can reach microorganisms that are inaccessible to scaling instruments and local antibiotic therapy [23]. This antibiotic therapy also has the potential to suppress any periodontal pathogenic bacteria colonizing the deep crevices of the tongue, as well as clinically nondiseased sites that could cause chronic reinfection. According to the American Academy of Periodontology, the patients who are likely to benefit from antibiotics are those for whom conventional mechanical treatment has proven ineffective, those suffering from acute periodontal infections (necrotizing periodontal disease and periodontal abscesses) or aggressive periodontitis, certain medically compromised patients, and patients who smoke [25]. Furthermore, periodontitis caused by *Aggregatibacter actinomycetemcomitans *often requires antibiotic treatment because this bacterium is found on all mucous membrane surfaces of the oral cavity and is capable of invading all soft tissues. These recommendations are in line with those of the French Health Products Safety Agency (AFSSAPS) [4].

The main approaches to systemic antibiotic therapy for periodontal treatment are based on monotherapy [26]. Amoxicillin with clavulanic acid is a broad-spectrum drug that shows low concentrations in gingival crevicular fluid (GCF). Metronidazole is an effective agent for treating refractory periodontitis involving *Porphyromonas gingivalis *and/or *Prevotella intermedia *[27, 28]; it is conducive to effective antibacterial concentrations in gingival tissues and GCF, but its oral administration seems to have little impact on oral and intestinal microflora. Tetracyclines (doxycycline and minocycline) are active against important periodontal pathogens such as *A. actinomycetemcomitans *[10, 29]; they also have anticollagenase properties and can reduce tissue destruction and bone resorption, although systemically administered tetracyclines reach relatively high concentrations in GCF. Clindamycin is effective in the treatment of refractory periodontitis and against Gram-positive cocci and Gram-negative anaerobic rods but should be prescribed with caution because of the risk of overgrowth of *Clostridium difficile, *which could result in pseudomembranous colitis [23]. Ciprofloxacin is effective against several periodontal pathogens, including *A. actinomycetemcomitans*; this antibiotic effectively penetrates the diseased periodontal tissues and can reach higher concentrations in GCF than in the serum [23, 30]. As periodontal lesions host a variety of periodontal pathogenic bacteria, it has become increasingly common to treat aggressive periodontitis using a combination of antibiotics. Such combinations include metronidazole and amoxicillin for *A. actinomycetemcomitans *infections and metronidazole and ciprofloxacin for mixed periodontal infections or for patients who are allergic to amoxicillin. Conversely, antagonistic effects are observed between certain antibiotics, for example, tetracyclines and certain *β*-lactams [23, 24, 26].

Several studies have been devoted to pharmacologic therapies that modulated host responses to periodontopathic bacteria. The purpose of host modulatory therapy (HMT) is to restore the balance of proinflammatory or destructive mediators and anti-inflammatory or protective mediators to that seen in healthy individuals. The only MMP inhibitor that has been approved for clinical use in the US, Canada, and Europe and tested for the treatment of periodontitis is subantimicrobial dose doxycycline or SDD. A number of studies have shown that the therapeutic effects of tetracycline antibiotics are to inhibit collagenolytic MMPs, to reduce connective tissue degradation, and to diminish bone resorption [31]. SDD, 20 mg twice daily for 2 weeks, significantly reduced collagenase activity in the GCF and gingival tissues of patients with adult periodontitis [32]. In another study, SDD was shown to improve certain clinical parameters (attachment level and probing depth) when administered to patients periodically over a 6-month period [33]. Evidence indicates that SDD also contributes to decreased connective tissue breakdown by downregulating the expression of proinflammatory mediators and cytokines (Interleukin-1 and tumoral necrosis factor-*α*) and increasing collagen production, osteoblast activity, and bone formation [7, 8, 11, 12, 18].

The aim of this study is to determine the effectiveness of systemic antibiotics as an adjunctive treatment in adult periodontitis therapy.

## 2. Materials and Methods

### 2.1. Search Strategy

An initial search was conducted of Elsevier, EBSCO, Wiley, PROQUEST, EJS, BIOMED, PubMed, Medline, and Ovid journals based on a combination of the following medical subject headings: periodontal disease, antibiotic therapy in periodontal treatment, antibiotic prophylaxis in periodontal treatment, antibiotic therapy in scaling and root planing, and periodontal microbiology.

### 2.2. Inclusion and Exclusion Criteria

Both English and non-English articles were included in the search of the literature. Articles that met the following criteria were reviewed and included in the meta-analysis.

1. Involves systemic antibiotic therapy (prophylactic, postoperatory, and HMT) as an adjunctive treatment in periodontal instrumentation (scaling and root planing).
2. Is described as a randomized double-blind clinical trial.
3. Includes a control group.
4. Includes clinical outcomes: gingival attachment level and periodontal probing depth.
5. Published between 2001 and 2011.

Studies that did not fulfill all of the aforementioned inclusion criteria were excluded from this paper. Clinical case reports, literature reviews, and in-vitro studies were also excluded.

### 2.3. Data Extraction

A database was used to retrieve information regarding study design, patient characteristics, sample size, control group, systemic antibiotic therapy (drug and dose), timing of administration, clinical outcomes, and *P* value.

### 2.4. Statistical Analysis

The data from the studies were analyzed by means of an analysis of variance (ANOVA) using a statistical software program (SPSS Statistics 19) and a meta-analytical program (Comprehensive Meta-Analysis V2) with a random-effect model. We determined that a random-effect model was more appropriate for this study given the variables and *P* values found in most of the clinical trials.

## 3. Results

### 3.1. Outcome Measure

Nine studies were included in the analysis (Table 1). All 9 reported changes in clinical attachment level (CAL) and changes in probing depth (PD); 7 reported percentage of bleeding on probing (BOP); 3 reported adverse effects; 2 reported gingival inflammation; 2 reported plaque index; 2 reported metabolic activity in gingival crevicular fluid based on the measurement of collagenase activity or terminal carboxytelopeptide of type 1 collagen (metabolic activity in GCF); 1 reported proportion of spirochetes; 1 reported antibiotic sensitivity of subgingival microflora (Table 2). A total of 864 patients were included in the final analysis: 441 were randomized to an antibiotic group and 423 to a control group, in 9 clinical trials that reported CAL gains and PD reduction as clinical outcomes. 380 patients were randomized to the SDD group, 61 patients to the amoxicillin-metronidazole group, and 423 to the control group. The clinical outcomes are summarized in Table 3.

The significance of differences between test and placebo groups in terms of numerical data was evaluated using an analysis of variance (ANOVA) for independent samples. All parameters showed a statistically significant difference at baseline. CAL gain and PD reduction in categories consisting of moderate (4–6 mm) and severe (≥7 mm) sites were significantly better in test subjects (*P* < 0.01, 95% confidence level, post hoc Bonferroni). The percentage of sites with CAL gain and PD reduction in categories consisting of moderate (4–6 mm) and severe (≥7 mm) sites was also significantly better (*P* < 0.01, 95% confidence level, post hoc Bonferroni). Golub et al. [7], Reinhardt et al. [15], Needleman et al. [16], and Griffiths et al. [17] were excluded from this analysis because they did not fulfill the criteria regarding changes in CAL and/or PD reduction.

Meta-analysis indicated that adjunctive systemic antibiotic therapy (SDD or amoxicillin and metronidazole) was statistically significant in CAL gain and PD reduction in chronic and aggressive periodontitis SRP (*P* < −0.01, 95% confidence level). See Tables 4, 5, 6, 7, 8, 9, 10, and 11.

## 4. Discussion

Periodontal diseases are caused by microorganisms that reside at or below the gingival margin. The best way to control these periodontal infections is to control the pathogenic species residing in these locations [19]. Ideally, periodontal therapy would reduce or eliminate the pathogenic species that cause and/or sustain periodontal diseases and maintain colonization by host-compatible species. Mechanical debridement of dental biofilm and the elimination of local irritating factors are the cornerstone of initial periodontal therapy, and systemic antibiotic treatment suppresses any reservoirs of periodontal pathogens that are not totally eliminated and which could potentially generate a chronic reinfection of the treated sites [23]. The host response to the plaque as a biofilm is equal or even more important than the bacterial biofilm when considering treatment targets and strategies for periodontitis [9]. Scaling and root planing (SRP) are the bases of nonsurgical therapy in the treatment of periodontitis. However, the results of this therapy are often unpredictable and are dependent upon many different factors [24].

The use of systemic antimicrobials as part of the therapy employed in the management of periodontal diseases has been debated for decades [24]. A meta-analysis by Haffajee et al. [26] reviewed 29 studies and reported that systemically administered antimicrobial agents provide a significant clinical benefit in terms of mean CAL gain. The included comparisons suggested a number of benefits of the adjunctive antimicrobial. Whilst there are sufficient data to suggest that antibiotics might help in the treatment of periodontitis, the optimum protocol of use has not been clearly defined. This lack of clear protocols of use may be due in part to the specific properties of biofilms, which make subgingival periodontal pathogens more difficult to target; therefore, the development of strategies specifically designed to treat the subgingival microflora, as a biofilm, is highly desirable. In the meantime, treatment strategies based on conventional therapies should be adapted to current knowledge on biofilms.

Amoxicillin with metronidazole is an antimicrobial combination that significantly improved short-term periodontal clinical outcomes [14, 26] thanks to its synergistic effect on the suppression of *A. actinomycetemcomitans* [14]. Herrera et al. [24] showed a statistically significant additional benefit of spiramycin on PPD change and amoxicillin/metronidazole on CAL change in deep pockets when they analyzed 25 controlled clinical trials in which systemically healthy patients with either aggressive or chronic periodontitis were treated with SRP in conjunction with systemic antimicrobials for a minimum of 6 months, as compared to SRP alone or being treated with a placebo.

Only two reports [7, 10] assess the microbiological characteristics of the bacterial pathogens involved in the subjects of their studies. It would be useful to evaluate, in randomized controlled double-blind placebo and control group clinical trials, the proportion of *P. gingivalis* associated with aggressive periodontitis [23] and of *A. actinomycetemcomitans* associated with chronic and aggressive periodontitis [34], as well as other specific associations among periodontal pathogens, in order to develop a prediction model based on the microbiological indicators of the metabolic activity of periodontal pathogens that would allow us to determine risk factors, progress, and the recurrence of periodontal disease, mediated by SDD and amoxicillin with metronidazole antibiotic regimens.

Clinical outcomes are a practical resource with which to establish periodontal status, though the reduction in numbers of subgingival pathogens, CAL improvement, and PD reduction are influenced by many other factors [11]. Severe periodontal disease has frequently been associated with smoking, diabetes, and polymorphism in the IL-1 gene [11]. Loss of bone mineral density in the skeleton (osteopenia/osteoporosis) has also been associated with loss of periodontal support [15]. Investigation groups study the microbiological relation between systemic conditions, local factors, and bacterial pathogen groups to elucidate treatment strategies aimed at maintaining periodontal stability.

When considering the various factors related to systemic antimicrobial usage in the treatment of periodontal diseases, adverse effects should always be taken into account, in particular, the side effects for individual patients and the increase in bacterial resistance, which is a major global public health problem. These factors should be considered when prescribing systemic antimicrobials, which should not be used routinely but rather in certain patients and under defined periodontal conditions [24, 35]. In a few cases, clinical trials have reported upset stomach, diarrhea, and general unwellness in patients (15%) treated with this regimen for 7 days [14]. Similarly, SDD studies have reported similar treatment-emergent adverse events among placebo and experimental groups, including events associated with the gastrointestinal and genitourinary tracts (infections) and the skin (rash and photosensitivity reactions) [12]. Various clinical studies using SDD have shown no difference in the composition or resistance level of the oral, fecal, or vaginal microflora, and these studies have shown no overgrowth of opportunistic pathogens in the oral cavity, gastrointestinal system, or genitourinary system. However, due to their non-antimicrobial proanaerobic and anticatabolic properties, SDDs are excellent candidate pharmaceuticals to simultaneously treat local (periodontitis) and systemic (osteopenia and osteoporosis) disorders [35–37].

Nevertheless, there were significant discrepancies in terms of study setting, case selection, and scientific rigor in the carrying out of these original studies. Efforts were made to use a random-effect model to increase the rigor of the statistical analysis. However, it cannot be stated that the outcome of the present study should be taken as a rigid guideline in clinical practice involving systemic antibiotic treatment in SRP. A well-designed randomized and placebo-controlled multicenter clinical trial is needed in order to be able to reach a definitive conclusion. Such definitive clinical trial should take into consideration known risk factors such as age, gender, smoking, and systemic conditions and have a standardized protocol for outcome assessments.

The results of this meta-analysis of randomized controlled double-blind clinical trials indicated that the host modulating agent, SDD, and the wide-spectrum antibiotic amoxicillin, in combination with the narrow-spectrum antianaerobic, represented by metronidazole, were both effective in improving CAL and reducing PD when administered as an adjuvant in the nonsurgical management of chronic and aggressive periodontitis. Finally, clinicians must not forget intense plaque-control techniques to establish better conditions in the patient's oral environment. Additional research continues to help determine an attractive approach for stimulating bone formation and engage global treatment strategies for reducing periodontal pathogens and so obtain better conditions for periodontal tissue regeneration.

## 5. Conclusion

In summary, the findings of this meta-analysis suggest that there is a role for systemic antibiotic treatment as an adjunctive therapy in the nonsurgical management of chronic and aggressive periodontitis. The host modulating agent, SDD, and the wide-spectrum antibiotic—in combination with the antianaerobic, represented by amoxicillin-metronidazole—were effective in improving clinical attachment level and reducing pocket probing depth. Each of these strategies offers advantages and disadvantages for clinical practice, and clinicians are encouraged to evaluate the evidence for each choice carefully and make an informed decision in the best interests of their patients.

## Figures and Tables

**Table 1:** Studies included in the analysis of the effectiveness of antibiotic treatment in nonsurgical periodontal therapy.

Year and authors	Periodontal disease	Study duration	Study groups
(1) Caton et al., 2000 [10]	Chronic periodontitis	9 months	SRP + SDD
			SRP + placebo
(2) Golub et al., 2001 [7]	Chronic periodontitis	36 weeks	SRP + SDD
			SRP + placebo
(3) Novak et al., 2002 [11]	Generalized severe periodontitis	9 months	SRP + SDD
			SRP + placebo
(4) Preshaw et al., 2004 [12]	Chronic periodontitis	9 months	SRP + SDD
			SRP + placebo
(5) Preshaw et al., 2003 [13]	Chronic periodontitis	9 months	SRP + SDD
			SRP + placebo
(6) Guerrero et al., 2005 [14]	Generalized aggressive periodontitis	6 months	SRP + amoxicillin-metronidazole
			SRP + placebo
(7) Reinhardt et al., 2007 [15]	Chronic periodontitis	24 months	SRP + SDD
			SRP + placebo
(8) Needleman et al., 2007 [16]	Chronic periodontitis	6 months	SRP + SDD
			SRP + placebo
(9) Griffiths et al., 2011 [17]	Generalized aggressive periodontitis	8 months	SRP + amoxicillin-metronidazole + placebo
			SRP + placebo + amoxicillin-metronidazole

SRP: scaling and root planing; SDD: subantimicrobial dose doxycycline. 20 mg/12 hours; Amoxicillin-metronidazole: 500 mg of each/8 hours/7 days.

**Table 2:** Outcomes measured in systemic antibiotic treatment as an adjunctive therapy in scaling and root planing.

Clinical and microbiological outcomes	Caton et al. 2000 [10]	Golub et al. 2001 [7]	Novak et al. 2002 [11]	Preshaw et al. 2004 [12]	Preshaw et al. 2008 [18]	Guerrero et al. 2005 [14]	Reinhardt et al. 2007 [15]	Needleman et al. 2007 [16]	Griffiths et al. 2011 [17]
(1) CAL gain	∗	∗	∗	∗	∗	∗	∗	∗	∗
(2) PD reduction	∗	∗	∗	∗	∗	∗	∗	∗	∗
(3) % of BOP	∗		∗	∗	∗	∗	∗	∗	
(4) Adverse events				∗		∗			
(5) Gingival inflammation index		∗	∗						
(6) Plaque index									∗
(7) Metabolic activity in GCF		∗						∗	
(8) Proportion of spirochetes	∗								
(9) Antibiotic sensitivity of microflora		∗							

CAL: clinical attachment level; PD: probing depth; BOP: bleeding on probing depth; metabolic activity in GCF gingival crevicular fluid.

**Table 3:** Clinical outcomes reported in randomized controlled trials evaluating the efficacy of adjunctive antibiotic treatment in SRP.

					CAL gain		Sites with PD reduction		% Sites with CAL gain		% Sites with PD reduction	
Authors	Periodontal disease	Study duration	Study groups	*n*	4–6 mm	≥7 mm	4–6 mm	≥7 mm	4–6 mm	≥7 mm	4–6 mm	≥7 mm
(1) Caton et al. [10]	Chronic periodontitis	9 months	SRP + SDD	90	1.03	1.55	0.95	1.68		22	47	22
			SRP + placebo	93	0.86	1.17	0.69	1.29	38	16	35	13
(2) Golub et al. [7]	Chronic periodontitis	33 weeks	SRP + SDD	27	−0.15							
			SRP + placebo	39	−0.8							
(3) Novak et al. [11]	Generalized severe periodontitis	9 months	SRP + SDD	10	1	1.78	1.2	3.02	29	15	48	26
			SRP + placebo	10	0.56	1.24	0.97	1.42	21	11	21	6
(4) Preshaw et al. [12]	Chronic periodontitis	9 months	SRP + SDD	107	1.27	2.09	1.29	2.3	58	33	.2	37
			SRP + placebo	102	0.94	1 .6	0.96	1.71	44	20	.5	21
(5) Mohammad et al., [8]	Chronic periodontitis	9 months	SRP + SDD	66	1.29	2.12	1.33	2.35	63	37	66	42
			SRP + placebo	76	1.01	1.55	1	1.74	45	20	47	22
(6) Guerrero et al. [14]	Aggressive periodontitis	6 months	SRP + AMOXL-METRO	20	0.5	1	0.4	1			30	55
			SRP + placebo	21	0.2	0.7	0.1	0.8			21	37
(7) Reinhardt et al. [15]	Chronic periodontitis	24 months	SRP + SDD	64						5	15	
			SRP + placebo	64						3.4	0	
(8) Needleman et al. [16]	Chronic periodontitis	6 months	SRP + SDD	16	0.65	1.4						
			SRP + placebo	18	0.4	0.98						
(9) Griffiths et al. [17]	Aggressive periodontitis	8 months	SAP + AMOXL-METRO + placebo	20							31	53
			SRP + placebo + AMOXL-METRO	21	0.3	0.7	0.4	0.9			27	49

CAL: clinical attachment level; PD: probing depth; SRP: scaling and root planing; SDD: subantimicrobial dose doxycycline, 20 mg/12 hrs; AMOXI-METRO: amoxicillin-metronidazole, 500 mg of each/8 hrs/7 days.

*Tooth sites were stratified according to the degree of disease severity (magnitude of PD) evident at baseline. Tooth sites with a baseline PD of 0 to 3 mm were considered normal, tooth sites with a baseline PD of 4 to 6 mm were considered mildly to moderately diseased; tooth sites with a baseline PD of >7 mm were considered severely diseased.

**Table 4:** Forest plot (meta-analysis, random effect model) indicating the cumulative effect sizes for clinical attachment level gain at sites with moderate periodontitis (4–6 mm).

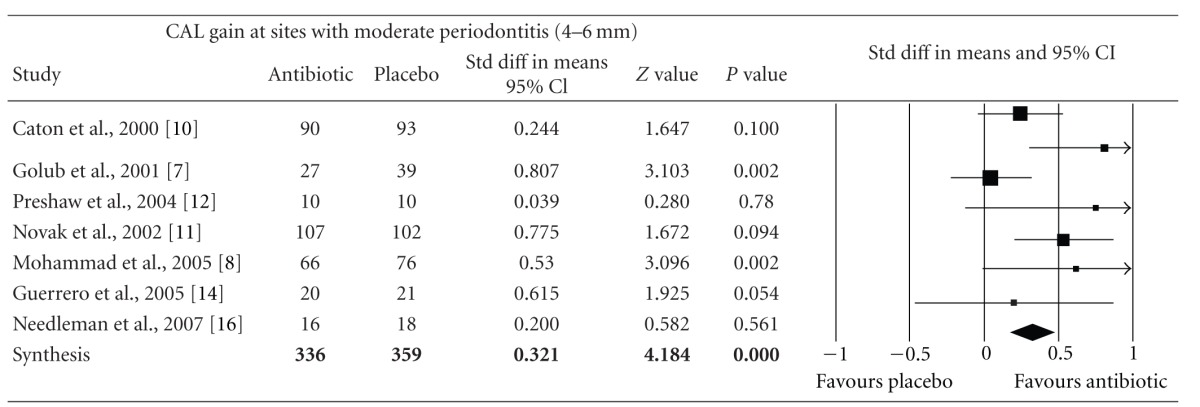

CAL: clinical attachment level, *P* < 0.05, confidence level 95%.

**Table 5:** Forest plot (meta-analysis, random effect model) indicating the cumulative effect sizes for percentage of sites with clinical attachment level gain at sites with moderate periodontitis (4–6 mm).

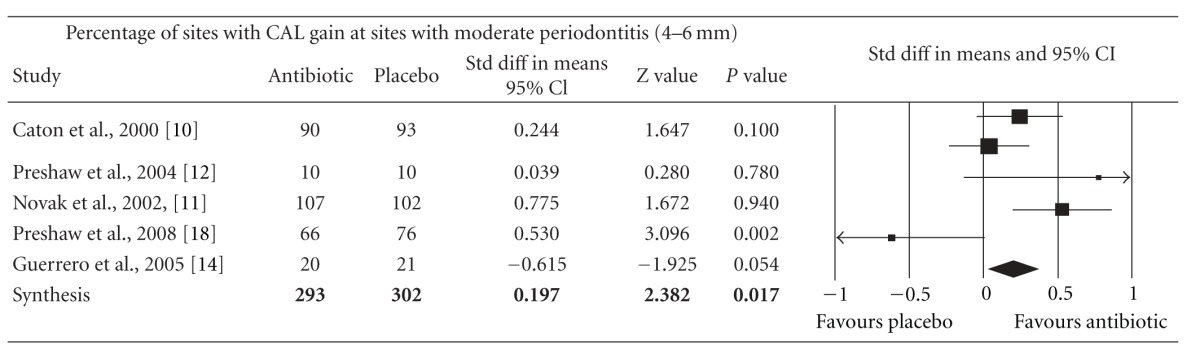

CAL: clinical attachment level, *P* < 0.05, confidence level 95%.

**Table 6:** Forest plot (meta-analysis, random effect model) indicating the cumulative effect sizes for clinical attachment level gain at sites with severe periodontitis (≥7 mm).

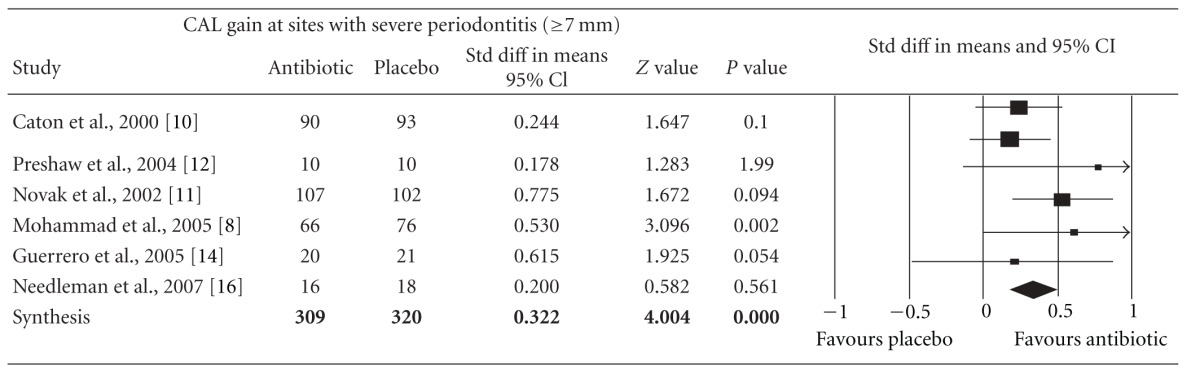

CAL: clinical attachment level, *P* < 0.05, confidence level 95%.

**Table 7:** Forest plot (meta-analysis, random effect model) indicating the cumulative effect sizes for percentage of sites with clinical attachment level gain at sites with severe periodontitis (≥7 mm).

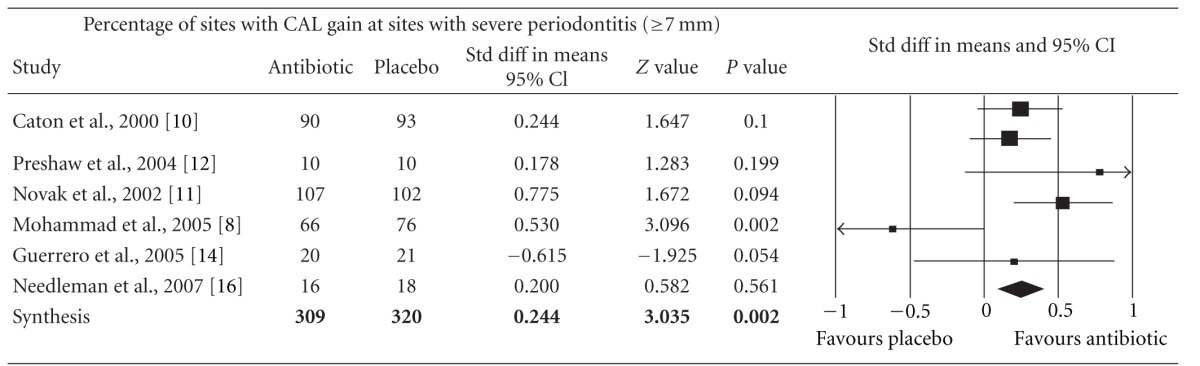

CAL: clinical attachment level, *P* < 0.05, confidence level 95%.

**Table 8:** Forest plot (meta-analysis, random effect model) indicating the cumulative effect sizes for probing depth reduction at sites with moderate periodontitis (4–6 mm).

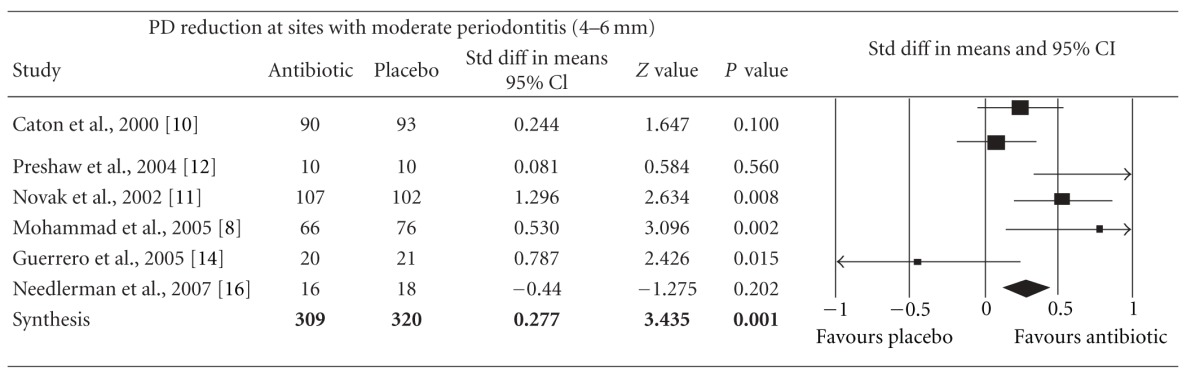

PD: probing depth, *P* < 0.05, confidence level 95%.

**Table 9:** Forest plot (meta-analysis, random effect model) indicating the cumulative effect sizes for percentage of sites with probing depth reduction at sites with moderate periodontitis (4–6 mm).

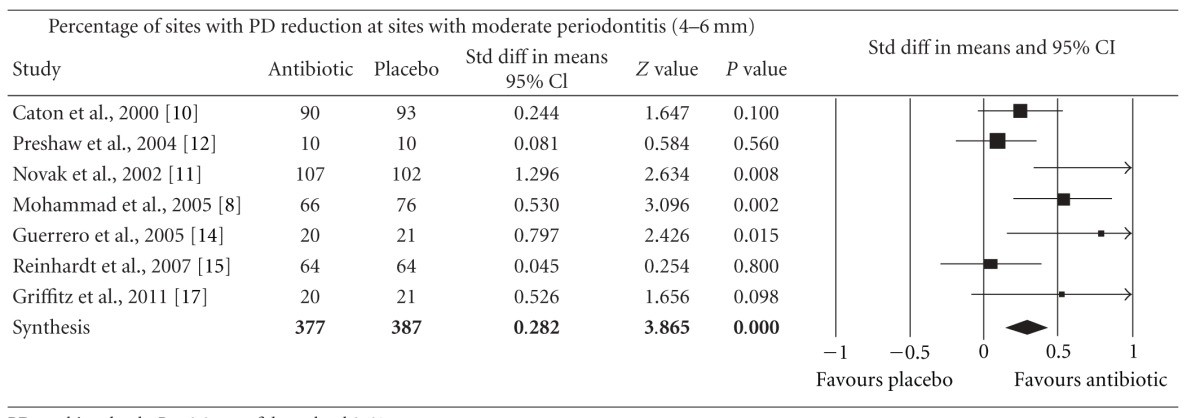

PD: probing depth, *P* < 0.05, confidence level 95%.

**Table 10:** Forest plot (meta-analysis, random effect model) indicating the cumulative effect sizes for probing depth reduction at sites with severe periodontitis (≥7 mm).

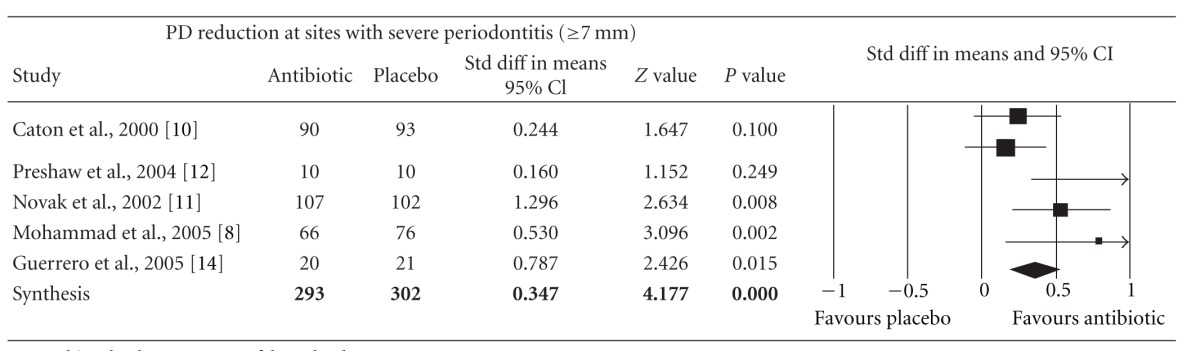

PD: probing depth, *P* < 0.05, confidence level 95%.

**Table 11:** Forest plot (meta-analysis, random effect model) indicating the cumulative effect sizes for percentage of sites with probing depth reduction at sites with severe periodontitis (≥7 mm).

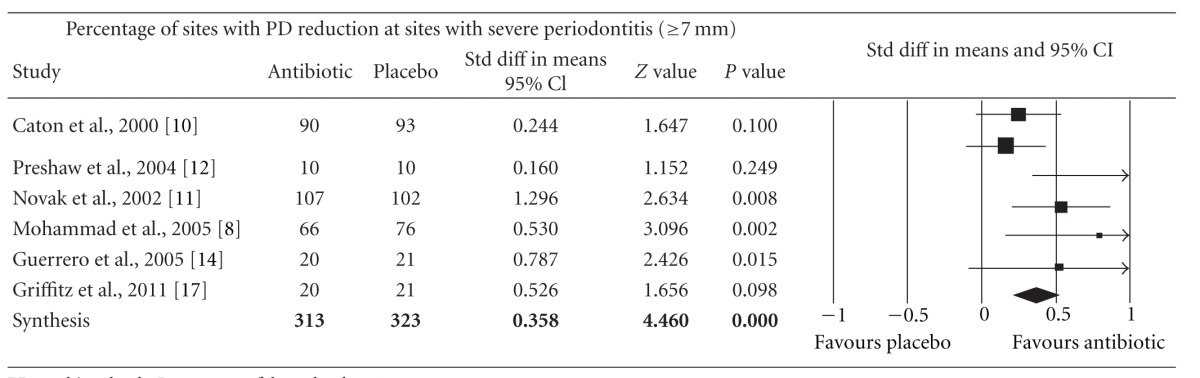

PD: probing depth, *P* < 0.05, confidence level 95%.
